# Factors Associated with Depression among Heart Failure Patients at Cardiac Follow-Up Clinics in Northwest Ethiopia, 2017: A Cross-Sectional Study

**DOI:** 10.1155/2019/6892623

**Published:** 2019-07-21

**Authors:** Kassahun Gebeyehu Yazew, Debrework Tesgera Beshah, Mohammed Hassen Salih, Tadele Amare Zeleke

**Affiliations:** ^1^Department of Medical Nursing, School of Nursing, College of Medicine and Health Science, University of Gondar, Gondar, Ethiopia; ^2^Department of Surgical Nursing, School of Nursing, College of Medicine and Health Science, University of Gondar, Gondar, Ethiopia; ^3^Department of Psychiatry, College of Medicine and Health Science, University of Gondar, Gondar, Ethiopia

## Abstract

**Background:**

Depression is a comorbid disorder in patients with heart failure and it is a major public health problem worldwide. Little is known about the depression among heart failure patients in low-income countries, while, in Ethiopia, none was studied.

**Objective:**

This study is to assess the prevalence of depression and associated factors among heart failure patients at cardiac follow-up clinics at Amhara Region Referral Hospitals, Northwest Ethiopia, 2017.

**Methods:**

A hospital based cross-sectional study was conducted between March 30, 2017, and May 15, 2017, G.C., by using a systematic random sampling technique to select 422 of 1395 HF patients. Structured interviewer-administered questionnaires and patient card review with a checklist that incorporates the PHQ-9 tool for depression measurement were used. The collected data were checked, coded, and entered into Epi-info version 7 and exported to SPSS version 20. Bivariate logistic regression at p-value <0.2 was exported to multivariate logistic regressions and p-value <0.05 was considered statistically significant.

**Results:**

A total of 403 were included with a response rate of 95.5%. Among the participants, 51.1% had depressive symptoms. Factors associated with depressive symptoms were poor self-care behavior 1.60 [AOR (95% CI=1.01, 2.55)], poor social support 1.90 [AOR (95% CI=1.16, 3.12)], being female 2.70 [AOR (95% CI=1.44, 5.07)], current smoking history 4.96 [AOR (95% CI=1.54, 15.98)], and duration of heart failure (>1 year) 1.64 [AOR (95% CI=1.04, 2.59)].

**Conclusions:**

Around half of the patients were depressive. The patients who had poor self-care behavior, were females, had poor social support, had a current history of smoking, and had duration of chronic heart failure >1 year need special attention. Therefore, all referral hospitals need efforts to focus on those problems and target improvements of depressive symptoms.

## 1. Introduction

Depression is the most common mental health condition in the general population [1], characterized by sadness, loss of interest or pleasure, feelings of guilt or low self-worth, disturbed sleep or appetite, feelings of tiredness, and poor concentration [2]. It is a common comorbidity in patients with heart failure (HF) [3]. In 2013, 61.7 million people suffered from HF worldwide, 55% of those at a severe stage [4], and it was a major public health problem worldwide [5].

According to the World Health Organization's estimate, depression and cardiovascular disease will be the two major causes of disability-adjusted life years by the year 2020 [6–8].

Depression in heart failure has become a major issue as the burden of heart failure has continued to increase. Consequently, many studies suggested that HF patients reporting depression had poorer outcomes in HF [9] and 29% higher medical costs than nondepressed patients [10] and it was found to be a strong and independent predictor of negative outcomes in patients with HF, such as cardiac events, readmission, and mortality [11].

Also, according to the Diagnostic and Statistical Manual of Mental Disorders IV, approximately 25% of the people with general medical conditions will become depressed during the course of their chronic condition [12]. Therefore, CVD and depression, profoundly impact the overall quality of life [13]. Poor mental health can result in poorer outcomes associated with other diseases like cardiovascular disease [14–16].

From Kerala, India, there was a need for psychological intervention to manage and control the symptoms of depression in cardiovascular diseases in each and every cardiology unit [17].

Just like other emerging countries, Ethiopia is also faced by the growing prevalence of the chronic noncommunicable disease (CNCDs), communicable disease, and injury which created a triple burden on the population and the health system. In fact, the former scholars explored that high magnitude of the CNCDs contains hypertension and other cardiovascular diseases in both rural and city parts of the country [18]. And this was because of the physical, psychological, and social consequences of depression, which negatively affects CHF and CHF symptoms generating depression, especially in those with high hazard [19]. Hence there is no data regarding depression and associated factors among heart failure adult patients in Ethiopia.

Based on one meta-analysis conducted in 2006 with the review of more than 36 studies, the prevalence of depression was over 20% of all HF patients, with twice the rates among patients with more severe heart failure. Patients with depression were also more than twice as likely to experience premature death or secondary events over time (REF) [20]. And also, in a different study, the prevalence of the depression among heart failure in the United States of America (USA) was 42.1% [21], United Kingdom 10–60% [19], Australia 52% [22], Japan 22.1% [23], Iraq 45.1% [24], Nigeria 48 % [25], and among admitted nonspecific CHF patients in Ethiopia 153 (54.6%) [26]. Due to the high prevalence in cardiac patients, researchers tried to control depression in heart failure patients on different drug trials [27].

Several factors that influence depression among patients with heart failure (HF) have been examined, such as gender, younger patients, living alone, poor social support, poor self-care behavior, poor knowledge on HF, disease severity, and lifestyle issues such as alcohol and cigarette smoking [20, 28–37].

A meta-analysis, regarding gender, stated that HF women experience more intense depression than men [34]. However, this was not in agreement with scholars who stated that men with HF were more likely to be depressed than women [37].

An integrative review from Tallahassee [31] and a longitudinal observational study from the United States [30] explained that social support positively impacts and influences the psychological well-being of those with HF.

A cross-sectional study from Brazil explained that self-care behavior was significantly associated with depression of the heart failure participants [29].

Prospective study from Australia described that smoking was an independent predictor of the depression among cardiac patients [36].

Meta-analysis study showed that New York Heart Association (NYHA) functional status was associated with the prevalence of depression [20]. Studies from Athens, Greece, revealed that heart failure over 1 year was significantly associated with a higher level of depression [35].

Therefore, this study aimed at assessing depression and associated factors among adults with HF at cardiac follow-up clinics in Amhara Region Referral Hospitals, Northwest Ethiopia.

## 2. Materials and Methods

### 2.1. Study Area and Population

A hospital based cross-sectional survey was employed in Amhara Region, Northwest Ethiopia, between March 30, 2017, and May 15, 2017. Amhara Region, Northwest Ethiopia, had three referral hospitals, namely, University of Gondar Teaching Referral Hospital, Feleghiwot Referral Hospital, and Deberemarkos Referral Hospital. Heart failure participants who were older than 18 years and have been on follow-up at least for 3 months, who visited the cardiac follow-up units of the Amhara Region Referral Hospitals during data collection period, were included in the survey. Critically ill patients were excluded from the study because, firstly, they could not give the written consent to participate in the research and, secondly, it is an ethical issue.

The sample size was determined by using a single population proportion formula considering the following assumptions: prevalence (p) of depression 50%, Z = standard normal distribution value at 95% confidence level of Z^a/2^ = 1.96, and margin of error (d) = 5%. This gave a sample size of 384 participants. Taking into consideration the 10% nonresponse rate, the total sample size was 422.

Among 1395 HF attendants in the three referral hospitals, Northwest Ethiopia, during the data collection period, 422 were recruited for the study. Study participants were included using a systematic random sampling procedure.

### 2.2. Data Collection

Data were collected by four trained BSc nurses using pretested interviewer-administered questionnaire and supervised by three MSc nurses and a principal investigator. The data collection instrument had different components: sociodemographic characters, behavioral attributes, knowledge attributes, and appraisal of the patient document for medical related characters using a checklist that was developed on the basis of various prior similar studies and further modified to include important variables of this study to fit the study area context [38–44]. The data were collected by using the Patient Health Questionnaire (PHQ-9) which ranges from 0 to 27 scores. In PHQ-9 tool there were four options (0=not at all, 1=several days, 2=more than half of the days, and 3=nearly every day) which were used to screen depression symptoms from the study participants [42] and in this study they were reliable with Cronbach's alpha=0.80.

### 2.3. Operational Definitions

The individual had poor self-care when she/he scored above the mean (33.65) of European Heart Failure Self-Care Behavior Score (EHFScBS) [39].

In this study, the individual had depressive symptoms when he/she scored ≥10 in PHQ-9 score [42].

The individual had poor knowledge when she/he scored below 6 out of 8 points in the knowledge scale [43, 45].

The individual had poor social support when she/he scored below the mean (51.12) of the Mini Social Support screening tool [44].

The questionnaire was initially developed in English and translated to Amharic (local language) and then back to English by different language professionals to check for connotation consistency and it was pretested in similar setting out of the study area using 5% of the total sample size. A necessary correction was done after the pretest (in the behavioral questionnaire during the pretest we considered only the lifetime of substance history, but, after pretest, we modified it to the current substance history of the participants and included it). Two-day training for data collectors and supervisors was given to make them clear with the tool, method of data collection, how to ask questions, the way of approaching participants, and how to rate answers. The collected data were checked cautiously on a daily basis for completeness, accuracy, and clarity by a supervisor and the principal investigator to control the overall events of the data collection.

### 2.4. Data Processing and Analyses

Data were analyzed using SPSS version 20. Bivariate analysis was done to see the association of each independent variable with the outcome variable at P<0.2. Potential confounders (important) variables were controlled and entered into a multivariate logistic regression model to identify the effect of each independent variable with the outcome variables. A p-value< 0.05 was considered statistically significant, and the AOR with 95 % CI was calculated to determine the association [46]. Finally, data were presented in tables, figures, and texts.

### 2.5. Ethical Considerations

They were obtained from Institutional Review Committee of School of Nursing, College of Medicine and Health Science, University of Gondar, Ethiopia. Prior to the data collection, a formal letter was written for each referral hospital for their willingness and informed consent was obtained from the study participants, after providing the necessary information on the aim, importance, and privacy issues of the study.

## 3. Results

### 3.1. Demographic Characteristics of Respondents

The current survey was employed to assess the prevalence of depression and associated factors among heart failure patients at cardiac follow-up clinics in Amhara Region Referral Hospitals. From a sample of 422, a total of 403 respondents were included in the study with a response rate of 95.5%. Out of the total participants, 19 (4.5%) were nonrespondents. The study consisted of 234 (58%) males. The mean and standard deviation (SD) for age of respondents were 52.3 years and 19.1 years, respectively, and 183 (45.4%) was above 57 age groups followed by age 48-57 years, 58 (14.4%). More than half of the participants, 260, (64.5%) were living in the urban area and the rest were in rural areas. Among the participants, 159 (39.5%) had poor self-care behavior and 91 (22.8%) of them had poor social support (Table 1).

### 3.2. Medical Related Characteristics

With the New York Heart Association, Class III 155 (38.5%), Class I 133 (33%), a clinical symptom of CHF ≥ one year of duration 221(54.8 %), and the majority of them (48.6%) were comorbid with hypertensive (Table 2).

### 3.3. Behavioral Characteristics of the Participants

The mean Heart Failure Self-Care Behavior score was 33.65. Among respondents 206 (51.1%) have been ever drinkers, 66 (16.4%) of them were current drinkers of alcohol. Among participants current and ever smokers were 20 (5%) and 64 (15.9%), respectively.

### 3.4. Knowledge Attributes

Out of the total 403 respondents, 291(72.2%) and 112 (27.8%) had poor and good knowledge of heart failure, respectively.

### 3.5. Prevalence of Depression among Heart Failure Patients

**Figure 1:**
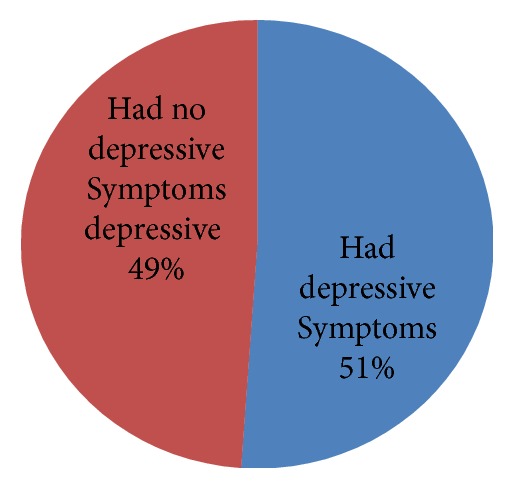
Prevalence of depression among adults with HF attending cardiac follow-up clinics in West Amhara Region Referral Hospitals, Northwest Ethiopia, 30 March to 15 May 2017 G.C (n=403).

The prevalence of depression among heart failure study subjects was 51%, who had symptoms, and 49%, who had no depressive symptoms (Figure 1).

### 3.6. Factors Associated with Depression among Heart Failure Patients

All independent variables were entered into the bivariate logistic regression model and variables with p ≤ 0.2 were entered into the multivariable analysis. After controlling possible confounding effects of other covariates, sex, self-care behavior, currently smoking, social support, knowledge, and duration of the heart failure had a significant association at 95% confidence level (Table 3).

## 4. Discussion

The prevalence of depression among heart disease patients was the most common disease we face. It occurs among the general population and often occurs in less developed countries. This study revealed that the prevalence of depression was 51.1% with the 95% CI (45.9%, 55.8%). This finding was in line with other studies carried out in the United Kingdom 10-60% [19], Australia 52% [22], Iraq 45.1% [24], Nigeria 48 % [25], and Ethiopia (54.6%) [26] among admitted nonspecific CHF patients.

On the other hand, the current study finding was higher than the study done in the United States of America 40.7% [21], Japan 22.1% [23], and Ethiopia, among the general population (5%) [47]. The variation might be due to the difference in study design, data collection tool, and sample size in study participants.

The current study was also identified as different factors that had an association with depression among CHF patients. With respect to gender, being female was significantly associated with depressive symptoms among CHF patients. Those females were 2.7 [AOR=2.70 (95% CI=1.44, 5.07)] times more likely to have depressive symptom than men, which was similar to the study done in Canada [34] and in Chicago [48]. The possible reasons for this might include a genetic predisposition and hormonal factors in women. On the contrary, the study showed that men with heart failure were more likely to become depressed than the females [37].

The other factor that had a significant association with depression was their self-care behavior. In this study, participants with poor SCB were about 1.60 [AOR=1.60 (95% CI=1.01, 2.55)] times more likely to have depressive symptom than those who had good self-care behavior. This study was supported by studies done in Brazil. Patients who practiced physical activity showed fewer symptoms of depression [29]. The possible justification for this one might be that the patient who had poor self-care behavior may be prone to depression due to the illness-related complication. And also the likely potential behavioral mechanism for the relationship between psychological factors and disease outcomes, such as poor quality of life, may be related to the effect of poor adherence to self-care and this again triggers the patient to be depressed [49].

The odds of depression were about 1.90 [AOR=1.90; (95% CI=1.16, 3.12)] times higher among individuals who had poor social support as compared to those who had good social support. This result was supported by USA [30] and Nigeria [25] studies. The reason might be that those patients who had poor social support were influenced by negative life stressors and it also plays a crucial role in the disturbance of the coping process for those with HF. So this phenomenon may, directly or indirectly, lead to depression.

In this study, the odds of depression were 1.67 [AOR=1.67 (95% CI=1.03, 2.69)] times higher among HF patients with poor knowledge when compared to those who had the knowledge. This result was similar to a Greek study [33]. The justification might be that patients who were not adequately informed about their health status or their treatment were more depressed compared to those who were sufficiently informed.

Another factor, the odds of depression, was 4.96 [AOR=4.96 (95% CI=1.54, 15.98)] times higher among current smokers as compared to those who had no smoking history. This finding was supported by studies done in Chicago [48] and Australia [36]. The possible reason may be that those who have an addictive problem like smoking are more prone to the risk of the mental problem. Just in case, cigarette smoke contains high amounts of oxidative free radicals [50] and those free radicals have a multifaceted effect on the oxidative defense system. Hence, it produces oxidative stress and, again, it is also implicated in the pathophysiology of depression [51].

The odds of depression were 1.64 [AOR=1.64 (95% CI=1.04, 2.59)] times higher among CHF more than one year of experience as compared to patients with duration of CHF less than one year since diagnosis. Supported by studies done in Athens, Greece [33], the possible reason might be that the level of depression is also associated with the duration of the disease, since patients who had been diagnosed for over 1 year experienced higher level of depression.

## Figures and Tables

**Table 1:** Demographic characteristics of participants attending cardiac follow-up clinics at Amhara Region, Northwest Ethiopia, 30 March to 15 May 2017(n=403).

Variables	Categories	Frequency	Percent (%)
Age in years	18-27	52	12.9
	28-37	46	11.4
	38-47	64	15.9
	48-57	58	14.4
	58^+^	183	45.4
Marital status	Single	49	12.2
	Married	268	66.5
	Divorced	31	7.7
	Widowed	55	13.6
Living Status	Alone	53	13.2
	With family	342	84.9
	With no family	8	2.0
Educational Background	Unable to read and write	183	45.4
	Can read and write	83	20.6
	Primary school	71	17.6
	High school and above	66	16.4
Occupation	Governmental employee	40	9.9
	Merchant	54	13.4
	Housewife	107	26.5
	Farmer	172	42.6
	Day laborer	30	7.3
Monthly income	586-1650	157	39
	1651-3145	119	29.5
	3146-5195	26	6.5
	5196-7758	8	2.0
	7759-10833	9	2.2
	>10833	1	0.2

**Table 2:** Medical related characteristics of the respondents attending cardiac follow-up clinics at Amhara Region, Northwest Ethiopia, 30 March to 15 May 2017 G.C. (n=403).

Variables	Categories	Frequency	Percent (%)
New York Heart Association class	II	39	9.7
	IV	76	18.9
	Beta-blocker	60	14.9
Types of Medications	Digitalis	53	13.2
	Diuretics	218	54.1
	Ca channel blockers	66	16.4
	No	161	40
Comorbidities	DM	17	4.2
	KD	8	2
	HTN+DM	6	1.5
	HTN+KD	5	1.2
	DM+KD	6	1.5
	Other∗	4	1
Duration of HF	< 1 year	182	45.2
	>1 year	221	54.8
Number of Hospitalizations	No	111	27.5
	Once	181	44.9
	Twice	49	12.2
	Three times	46	11.4
	Four times and above	16	4.0
	Four times and above	16	4.0
	Four times and above	16	4.0

*Note:* ∗Other- liver diseases.

**Table 3:** Bivariable and multivariable logistic regression output for factors associated with depressive symptoms among HF patients at cardiac follow-up clinics in Amhara Region, Northwest Ethiopia, 2017 (n=403).

Variables		Depression		COR [95%C.I.]	AOR [95%C.I.]	P-value
		Yes	No			
*Sex*	*Female*	*108*	*61*	*2.46(1.58,3.53)*	*2.70(1.44, 5.07)*	*0.01*
	Male	98	136	1	1	
*self-care behavior*	*Poor*	*96*	*63*	*1.86(1.24, 2.81)*	*1.60(1.01, 2.55)*	*0.04*
	Good	110	134	1	1	
*SS*	*Poor*	*56*	*36*	*1.67(1.04, 2.68)*	*1.90(1.16, 3.12)*	*0.01*
	Good	150	161	1	1	
*Knowledge *	*Poor*	*159*	*132*	*1.67(1.07, 2.59)*	*1.67(1.03, 2.69)*	*0.03*
	Good	47	65	1	1	
Living Status	Alone	32	21	1.54(0.86, 2.78)	0.62(0.61, 2.56)	0.18
	Not alone	174	176	1	1	
Occupation	NGE	190	173	1.65(0.85, 3.20)	1.64(0.78, 3.44)	0.19
	GE	16	24	1	1	
*Currently smoking*	*Yes*	*16*	*4*	*4.06(1.33, 12.37)*	*4.96(1.54,15.98)*	*0.01*
	No	190	193	1	1	
*Duration of CHF *	*>1 year*	*125*	*96*	*1.62(1.09, 2.41)*	*1.64(1.04, 2.59)*	*0.03*
	< 1 year	81	101	1	1	

*Note: NGE: *nongovernmental employment*, GE: *governmental employment, SS: social support, *COR:* crude odds ratio*, AOR:* adjusted odds ratio, *CI:* confidence interval *1. Living Status;* not alone: living with family+ living with nonfamily. These were merged because those living with nonfamily were few in number.

*2. Non*governmental: merchant+ housewife+ farmer+ daily laborer. These were merged because all are private occupations.

## Data Availability

The data used to support the findings of this study are available from the corresponding author upon request.

## Abbreviations

CHF: Chronic heart failure
CNCDs: Chronic noncommunicable disease
DM: Diabetes mellitus
ESC: European Society of Cardiology
HF: Heart failure
HFSCB: Heart failure self-care behavior
HTN: Hypertension
KD: Kidney disease
NYHA: New Work Heart Association
SCB: Self-care behavior
SD: Standard deviation
USA: United States of America.

## Additional Points

Because of the fact that the cross-sectional study design was used, it did not show the cause and effect relationship between the independent and dependent variables. The PHQ-9 is a screening tool. Therefore, it did not show the clinical diagnosis of the participants.
